# A Magnetic Resonance-Relaxometry-Based Technique to Identify Blood Products in Brain Parenchyma: An Experimental Study on a Rabbit Model

**DOI:** 10.3389/fvets.2022.802272

**Published:** 2022-05-31

**Authors:** Francesca Del Signore, Massimo Vignoli, Leonardo Della Salda, Roberto Tamburro, Andrea Paolini, Ilaria Cerasoli, Matteo Chincarini, Emanuela Rossi, Nicola Ferri, Mariarita Romanucci, Ilaria Falerno, Francesco de Pasquale

**Affiliations:** ^1^Veterinary Faculty, University of Teramo, Teramo, Italy; ^2^Clinica Veterinaria Borghesiana, Rome, Italy; ^3^Istituto Zooprofilattico Sperimentale dell'Abruzzo e del Molise Giuseppe Caporale, Teramo, Italy

**Keywords:** magnetic resonance, relaxometry, low field scanners, intracerebral hemorrhage, hierarchical clustering, receiver operating curve analysis

## Abstract

Magnetic resonance relaxometry is a quantitative technique that estimates T1/T2 tissue relaxation times. This has been proven to increase MRI diagnostic accuracy of brain disorders in human medicine. However, literature in the veterinary field is scarce. In this work, a T1 and T2-based relaxometry approach has been developed. The aim is to investigate its performance in characterizing subtle brain lesions obtained with autologous blood injections in rabbits. This study was performed with a low-field scanner, typically present in veterinary clinics. The approach consisted of a semi-automatic hierarchical classification of different regions, selected from a T2 map. The classification was driven according to the relaxometry properties extracted from a set of regions selected by the radiologist to compare the suspected lesion with the healthy parenchyma. Histopathological analyses were performed to estimate the performance of the proposed classifier through receiver operating characteristic curve analyses. The classifier resulted in moderate accuracy in terms of lesion characterization.

## Introduction

In order to investigate encephalic disorders in companion animals, MRI is considered the gold standard imaging technique (1, 2). These studies are based on signals weighted on the actual longitudinal (T1) and transverse (T2) tissue relaxation times, are typically qualitative. To overcome this limitation, a quantitative technique able to provide the actual T1/T2 values has been developed, called magnetic resonance relaxometry (MRR) (3, 4). A complete model linking relaxation times to the tissue microstructure remains to be firmly established. However, they can still be related to disease-induced tissue changes, developmental plasticity, and other biological processes. For this reason, MRR studies potentially offer a more detailed tissue characterization than the conventional MR examination. In human medicine, several MRR applications have been investigated in the clinical field. For example, this technique yielded promising results in identifying neoplastic lesions in the brain and in monitoring the response to chemotherapy (5–9). Many reports are also available on the application of this technique in epileptic patients, with the result of optimizing and enhancing lesion depiction (10–15). In the case of neurodegenerative disorders, MRR improved the detection of areas involved in early degeneration (16–18). A general review of this technique can be found in prior publications (3, 4). Recently, advanced MRR techniques based on susceptibility-weighted imaging (SWI) and magnetization transfer (MT) have been developed. However, the widespread use of these techniques is limited by the requirement of high field scanners and the adoption of biophysical models (see Discussion). Concerning MRR in animal models, most studies have been performed on rats, mainly investigating neoplasms (19, 20). Furthermore, MRR has been useful in characterizing hippocampal involvement in epileptic patients (21) and in detecting spontaneous brain hemorrhages (22). However, most animal studies lack accurate histopathological validation and are typically performed with high field scanners. These are characterized by a high spatial resolution and signal-to-noise ratio (SNR) (21). Translating such protocols in the clinical field might be challenging in case high field scanners are not available. Low field scanners have been widely used in veterinary practice (23) and nowadays are still in use in many veterinary facilities, but they have a low spatial resolution and SNR. Currently, the diagnosis of encephalopathies is typically based on standard T1w/T2w images, and small lesions can be missed. A correct diagnosis often relies on the radiologist's expertise in case of mild lesions, hepatic encephalopathy, distemper encephalitis, or small hemorrhages (24, 25). To overcome the above limitations, MRR is a promising method which can be successfully used using low field scanners. In a preliminary study, we have shown the potential of such a protocol implemented on a 0.25 T scanner and tested on phantom and real data (22). In the current work, we build upon those results to focus on experimentally induced brain lesions in rabbits. This model utilizes brains of similar size to canine and feline species so the protocol might be suitable for them. Animals with a smaller brain were excluded due to the prohibitive SNR of the MR scanner. The aim of this study was, first, to compare conventional T1w/ T2w low field images with MRR concerning the visibility of lesions (size and margins) and accuracy of the location. The second aim was to test the performance of the developed classifier to differentiate between healthy and abnormal tissue. We hypothesize that MRR might outperform conventional low field T1w/T2w sequences concerning lesion identification/ visibility, location, and extent.

## Materials and Methods

### Cerebral Hemorrhage Model in Rabbits

Sixteen New Zealand White male rabbits (*O. Cuniculus*) (3.2 ± 0.5 kg) were included in this study, which was carried out following recommendations from the National Committee for Animal Welfare (protocol N° 726/2019-PR). The number of subjects was dictated by this committee, and it was chosen to reach a larger size effect than in a previous study (26) where high field scanners (9.4 T) were employed. The software G^*^Power (27) estimated 13 subjects for a statistical power of 80% and a significance level of alpha = 0.05. Three additional subjects were added since we expected a mortality rate of 20%. Only male animals were included following committee recommendations. All subjects were kept under the supervision of trained personnel and received appropriate care according to national guidelines Eight hours before the procedure, each rabbit received meloxicam (1.5 mg/kg) (Metacam, 1.5 mg/ml, Boehringer Ingelheim Div. Veter) intramuscularly (IM) to obtain multimodal analgesia integrated with morphine (1 mg/kg) (morphine chloride, 10 mg/mL, S.A.L.F. S.p.a) and midazolam (0.5 mg/kg) (Dormicum®, 5 mg/ml, Roche Pharma, Switzerland) for premedication before the procedure. Each rabbit received topic administration of EMLA cream 5% ® (Aspen Pharma Trading Limited) 60 min before placement of auricular venous and arterial catheters (22–24 G 2.5 cm) to properly administer fluids and drugs as needed, and to measure blood pressure, respectively. Induction was performed with alfaxalone (1–4 mg/kg) (Alfaxan®, 10 mg/ml, Jurox, UK) intravenously (IV), and general anesthesia was maintained with isoflurane in oxygen 100% after applying a cuffed orotracheal tube (2–3–5 mm ID) under endoscopic guidance. The cerebral hemorrhage model was instituted by autologous blood injection, with 0.15 ml of blood extracted from the auricular vein. The rabbit was placed in sternal recumbency; a 1.5 cm midline sagittal skin incision was made with a #10 scalpel blade, and the subcutaneous tissue was dissected to visualize the calvarial intersection between the coronal and the caudal suture that was set as starting point. A 2.0 mm bone defect was manually created on the left calvarium with a Synthes-Colibrì 2 surgical drill using a Synthes drill bit (diameter 2 mm). The drilling starting point was 3–5 mm to the left of the coronal suture and caudal to the sagittal suture (28) (Supplementary Figure 1). The drilling was oriented perpendicularly to the parietal bone surface. A stopper was applied 4 mm below the tip of the drill to avoid any potential brain damage. Blood was then manually injected into the left hemisphere of each subject with a 1 ml syringe and a 25 G-1.6 cm needle oriented perpendicularly to the parietal bone surface to allow comparison between the two hemispheres in the same acquisition. To avoid crossing the entire brain section (around 2–3 cm) with the needle, the depth of injection was set to 0.5 cm. The rabbit was then carefully moved to the MR scanner. After the MR image acquisitions, each subject was euthanized according to national guidelines, and the brains were promptly fixed in 10% neutral buffered formalin for histopathological investigations (see below). Thirteen out of sixteen subjects were included in the study since three rabbits died. The autopsy did not show any fatal lesions, and death was attributed to the depressant effects of anesthetics (29).

### MRI Acquisition and Relaxometry Protocol

Magnetic resonance data were acquired using an Esaote Vetscan Grande scanner operating at 0.25 T and equipped with a Coil 4 (Esaote S.PA, Genova, Italy). The conventional data consisted of Spin Echo (SE) T1w (acquisition time = 5 min) and Fast SE T2w sequences in transverse (acquisition time = 6 min) and sagittal (acquisition time = 4 min) planes. Acquisition parameters are listed in Table 1. Based on these acquired images, a single transverse brain slice was selected to perform both T1 and T2 relaxometry. This slice was selected to contain the suspected localization of the lesion in the transverse plane. When the lesion was not clearly identifiable in this plane, sagittal sequences were used to localize the lesion. T1 relaxometry data were acquired through an *ad-hoc* protocol based on repeated acquisitions of SE T1w images corresponding to a variable TR, namely TR = [50,120, 200, 300, 400, 500, 600, 650, 750, 129, 850, 900, 950, and 1,000] ms [corresponding to acquisition times = (28, 62, 95, 115, 150, 180, 205, 245, 300, 321, 369, and 390) s]. This set of TR values had been optimized in a previous study (22). T2 relaxometry data were obtained by means of FSE T2w images repeatedly acquired with a variable TE, namely TE = [28, 75, and 136] ms [corresponding to acquisition times = (30, 95, and 115) s]. To account for small head movements during the acquisition, all acquired images were co-registered with the T1w images through an affine transformation using a standard approach (30). Using this approach, the acquired images, T1/T2 maps, and clustering results are aligned in the subject space. The complete acquisition procedure lasted about 45 min per subject.

**Table 1:** MRI acquisition parameters: settings for T1w–T2w transverse and sagittal sequences.

	**T1w Transverse**	**T2w Transverse**	**T2w Sagittal**
TR	400 ms	2,660 ms	2,170 ms
TE	18 ms	100 ms	100 ms
Slice thickness	3 mm	3 mm	3 mm
Gap	0 mm	0 mm	0 mm

### Analysis Pipeline

The proposed approach consists of a classification procedure that combines both the information from T1 and T2 relaxation times. The analysis pipeline is schematized in Supplementary Figure 2.

#### Estimation of T1 and T2 Maps

To estimate T1/T2 maps, the acquired MR signals were fitted through a two-parameter model using an unconstrained minimization algorithm based on a derivative-free method (31, 32). An expert radiologist selected a set of *n* voxels from the obtained T2 maps, representing regions of interest (ROIs) of suspected lesions for each subject (Supplementary Figure 2A). Then, the algorithm automatically extracted additional *n* regions analog to the ones selected in the contralateral hemisphere (Supplementary Figure 2A). The set of *2n* voxels represents the input of the classification algorithm. This allowed us to compare the selected ROIs with corresponding areas of the contralateral hemisphere to obtain subject-specific results, avoiding the normalization into an average-based brain template. The value of *n* was variable depending on the extent of the suspected lesion. To compare conventional MRI with MRR we used the following approach. The radiologist, not blinded to the site of injection, first evaluated the T1w and then T2w images of each rabbit. Then, he considered the respective T1 and T2 maps in order to assess if the lesion was detectable. The qualitative evaluation by the radiologist was conducted to classify a lesion as: “not visible,” “barely visible” or “clearly visible.” This classification was based on the following criteria:

- “Not visible” if the radiologist was not able to assess the location of the lesion in any plane of the acquisition;
- “Barely visible” if the radiologist was able to assess the location of the lesion, without being confident in the assessment of the extent and the margins of the lesion due to low contrast with the surrounding parenchyma;
- “Clearly visible” if the radiologist was able to assess the location, the extent, and margins of the lesion. In these cases, the radiologist also defined the shape of the lesion as linear, irregular, or circular.

Based on these criteria, the radiologist judged MRR as more accurate if the number of lesions classified as “clearly” or “barely” was higher than on conventional MRI. A statistical test was not performed at this stage.

#### Hierarchical Clustering

A hierarchical clustering driven by the T1 relaxometry patterns (Supplementary Figure 2B) was used to distinguish hemorrhagic vs. healthy tissues (Supplementary Figure 2C). At this stage, we adopted the original relaxometry pattern for every voxel to describe the return of the MR signal to the baseline. Specifically, the hierarchical clustering was run with the Euclidean distance and an unweighted average as linkage criterion (33). These two parameters relate to how the similarity among clusters is assessed. For every hierarchy, the clustering will link the pair of clusters showing the smallest euclidean distance computed among their centroids (linkage). The final output of the hierarchical clustering is a dendrogram showing the hierarchical evolution from a set of completely separated observations to the final single cluster, (Supplementary Figure 2C). At this stage, in order to choose the optimal number of clusters for every output, we computed the average cluster silhouette corresponding to a variable number of classes (from 2 to 5), for details (34). We assured that, in all subjects, the silhouette was maximized corresponding to two classes, i.e., a binary classification. In what follows, the cluster of voxels whose majority was in the healthy hemisphere was labeled as “healthy” (H), the other cluster as “pathological” (P). The control hemisphere was always the right since we induced hemorrhages only in the left hemisphere. The reported analyses were performed through in-house developed codes in MATLAB (MATLAB and Statistics Toolbox Release 2015b, The MathWorks, Inc., Natick, Massachusetts, USA). These codes can be run either under a MATLAB license or exported as stand-alone applications. In terms of computational time, with an Intel(R) Core (TM) i7-8550U CPU @, 1.8 GHz, 2. GHz running with 16 GB of RAM, the overall analyses (T1/T2 map estimation and hierarchical clustering) take ~20 min. In this study, we performed these steps after MR sessions. However, if codes were optimized and run on more powerful machines, these analyses could be done “online” during the MR acquisition.

#### Histopathological Examination

**Figure 1:**
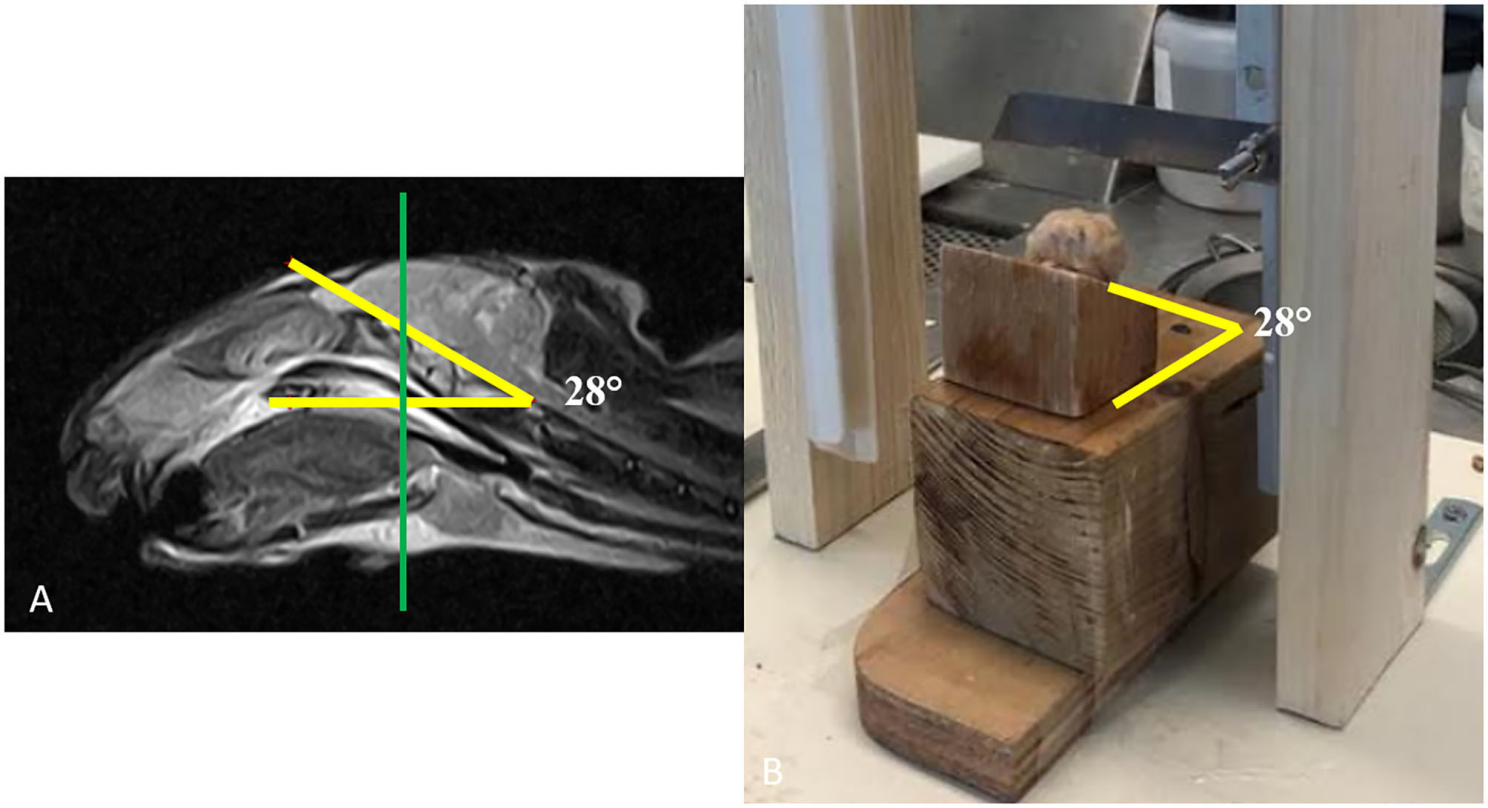
Operative procedure for brain cuts. **(A)** The inclination of the brain obtained from the T2w sagittal images and **(B)** the same inclination as in **(A)** has been reproduced with wooden wedges to be used with a small cutting tool specifically prepared for the experiment.

Brains were cut with a small cut system, purpose-built for the experiment (see Figure 1). To respect the same brain angle inclination measured from MRI images (Figure 1A), each brain was positioned on a wooden wedge (Figure 1B). Great care was taken to orient the cut parallel to the induced lesion. Transverse 4/5 mm thick serial sections of all fixed brains were also obtained and photographed (tissue blocks representative of all induced hematoma areas, as well as from the ipsilateral and/or contralateral nervous tissues, particularly frontal Cerebral Cortex, Corpus Callosum, and Caudate Nucleus). Paraffin-embedded brain 4 μm thick sections were routinely processed for histology and stained with hematoxylin-eosin. For the acquisition of the histological serial images, a Leica DMRE light microscope was used and the evaluation of the margins of the lesions was carried out using the image analysis software LAS X Measurements (Leica microsystem). The low magnification images were obtained with an objective Leica M PL APO MACRO 1x/0.035-0.006.

#### Validation of the Proposed Approach With Histopathological Analyses

To validate the proposed approach, the classification results were compared to the histopathological findings as follows. The classifier performance was assessed through a receiver operating characteristic (ROC) analysis where the gold standard was represented by the histopathological results (Supplementary Figure 2D). First, based on the histological analyses, the contours of the hemorrhagic tissues were extracted. In what follows, we will refer to this contour as the “true lesion contour.” Then, these images were co-registered to the anatomical MRI images by the radiologist and pathologist. The co-registration consisted of several steps (see Supplementary Figure 3, where we show the analysis for one of the reported subjects). The general idea is that the histopathological analyses were driven by the acquired MR images. To select the brain cut corresponding to the same transverse MR slice, we proceeded as follows. During the MR acquisition, when selecting the slice, we estimated the angle of inclination for every subject. Then, we built a wooden wedge reproducing such an angle (see Figure 1B). In this way, once the brain was extracted from the skull and placed on its wedge, the cut would be performed at the correct inclination. Once inclined, the cutting tool was oriented perpendicularly to the midline of the brain (to select a transversal slice as in the MRI). The tool was placed on the site of the injection (clearly visible on the cortex, see Supplementary Figure 3A). We chose the same location to select the slice during the MRI acquisition. Once we established the same inclination, orientation, and positioning, the anatomical cut would have closely matched the MR transverse slice. In Supplementary Figure 3B we show the brain cut, that we will refer to as a “macro image” (MI). All the histological analyses were performed on samples extracted from areas within MI. For this reason, the histological results obtained at different scales were aligned to MI (see Supplementary Figures 3C,D). Based on these analyses the pathologist estimated the final contour of the lesion and reported on MI (see Supplementary Figure 3E, white line). To co-register MI with the acquired T2w images (Supplementary Figure 3F), we adopted the Registration Estimator (part of the MATLAB Image Processing Toolbox). We performed a two-step registration. First, we applied a “similarity” transformation, which is a conformal mapping including rotation, translation, isotropic, and reflection (35). This was estimated by means of a regular step gradient descent algorithm (36), whose parameters are reported in Supplementary Figure 3G. This approach outperformed other possible strategies, available in the registration tool, such as an affine transformation. Second, to account for the local effect of the shrinkage, we applied a non-rigid transformation. In Supplementary Figure 3H we report the estimated similarity matrix (top) and the displacement fields of the non-rigid transformation (bottom). All the initial parameters of the involved algorithms were manually optimized by the radiologist and pathologist based on the quality of the obtained results. In Supplementary Figure 3I we show the initial point, the affine registration, and the final co-registration results. The MI image (Magenta) is overlaid on the T2w image (gray). Once the co-registration was completed, we adopted the lesion contour, mapped into the T2w image, as our gold standard (see Supplementary Figure 3I, yellow line). This allowed computation of the following parameters for every lesion. The true positives (TP), as the number of voxels correctly classified as pathological (red voxels in the figures), i.e., voxels falling within the “true lesion contour.” The false negatives (FN), as the number of voxels classified as healthy falling inside the “true lesion contour.” The false positives (FP), as the number of voxels classified as pathological that fell outside the “true lesion contour” and the true negatives (TN) as the number of voxels classified as healthy (yellow in the figures) falling outside the “true lesion contour.” Based on these parameters, the accuracy (ACC), true positive rate (TPR), and false-positive rate (FPR), according to the available literature (37), are defined as:


$$\begin{array}{l} \text{ TPR}=\text{TP}/(\text{TP}+\text{FN})\\ \text{ FPR}=\text{FP}/(\text{FP}+\text{TN})\\ \text{ACC}=(\text{TP}+\text{TN})/(\text{TP}+\text{TN}+\text{FP}+\text{FN}). \end{array}$$

Of note, these parameters allow us to account for the real size of the lesion. In case of a large lesion, if MRR correctly classifies only a part of it, this will increase FN. Thus, TPR, being normalized by (TP+FN), will be smaller. On the other hand, if the lesion is small and the MRR covers the entire lesion but overestimates it, this will increase FP. In this case, FPR, being normalized by (FP+TN), will decrease. These analyses were performed on the whole set of *2n* voxels comprising regions from both hemispheres. We included regions from the “healthy” hemisphere too since the classifier does not have any prior constraint on the voxels' locations. Thus, it might classify (erroneously) these voxels as pathological (FP will increase in this case). This is an important control for the performance. If the classifier finds a positive in the healthy hemisphere, it would be more severe than if it was near the lesion where the MR signals might be very similar to the pathological ones. Based on these parameters, we computed the ROC curves and the corresponding area under the curve (AUC) that can be interpreted as follows:

AUC = 0.5 the test is not informative; 0.5 < AUC ≤ 0.7 the test is not accurate; 0.7 < AUC ≤ 0.9 the test is moderately accurate; 0.9 < AUC <1 the test is highly accurate; AUC = 1 perfect test (32, 38).

Two hundred seventy-two ROC analyses were performed using an in-house developed code in MATLAB (MATLAB and Statistics Toolbox Release 2015b, The MathWorks, Inc., Natick, Massachusetts, USA).

## Results

### Comparison of Conventional MRI vs. MR Relaxometry

**Figure 2:**
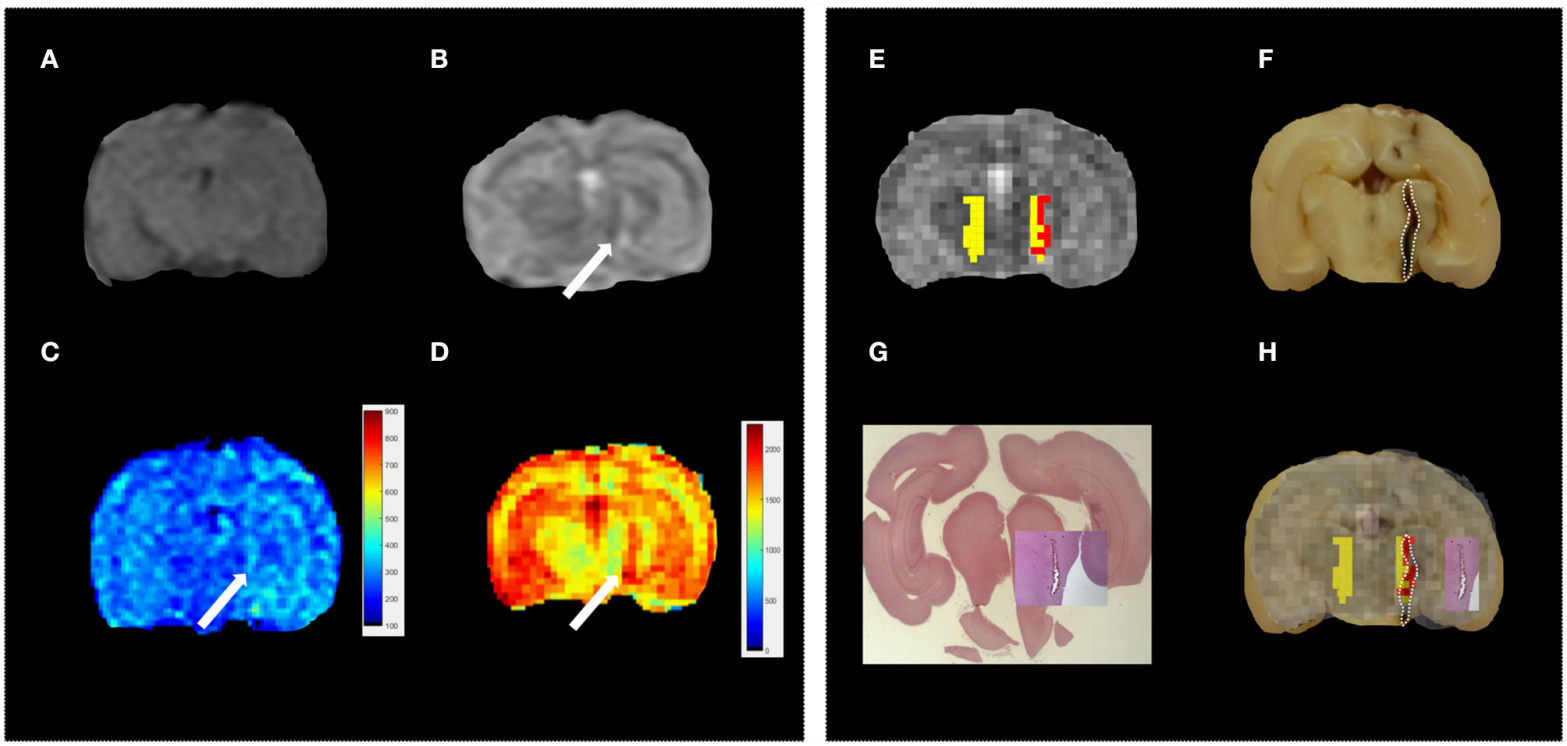
Standard vs. MRR analyses and histopathological validation. Example 1. In all panels, the left brain hemisphere is shown on the right part of the image. **(A)** T1w transverse image of the brain; the lesion has been classified as “not visible,” **(B)** T2w transverse image of the brain; the lesion appears ill-defined, hyperintense, and linearly shaped in the left thalamus (white arrow), **(C,D)** T1 and T2 maps, respectively. A hyperintense linearly shaped area is visible in both maps (white arrow), **(E)** the output of the hierarchical cluster overlaid on the T2 map. Voxels classified as “pathological” and “healthy” parenchyma are reported in red and yellow, respectively, **(F)** the results of gross anatomy. From the photograph of the brain section, the contour of the hemorrhagic lesion characterized by a linear shape that mainly involves the left thalamus (white arrow) has been delineated (white dotted line), **(G)** Histopathology results with the overall brain section obtained from the microscope with the hemorrhagic lesion shown in greater detail superimposed on the overall section, and **(H)** the MRR classification compared to both gross anatomy and histopathology: voxels classified as pathological (red) fall inside the estimated true lesion contour.

**Figure 3:**
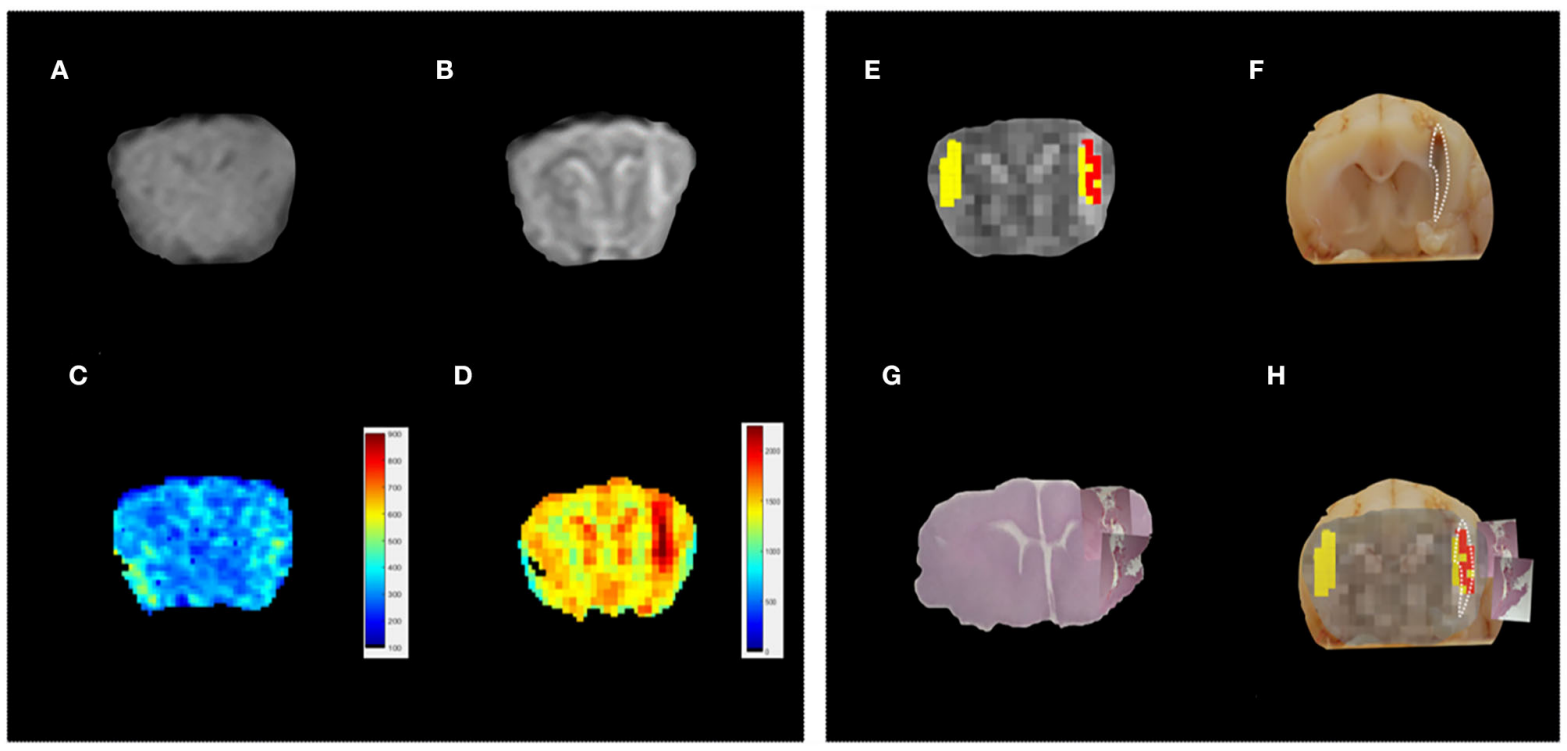
Standard vs. MRR analyses and histopathological validation. Example 2. In all panels, the left-brain hemisphere is on the right part of the image. **(A)** T1w transverse image of the brain; the lesion has been judged “not visible” by a board-certified radiologist. **(B)** T2w transverse image of the brain. Also, in this case, the lesion appears ill-defined, hyperintense, and linearly shaped in the Nucleus Caudatus (white arrow), **(C,D)** T1 and T2 maps, respectively. A hyperintense linear-shaped area is visible in both maps (white arrow), **(E)** The output of the hierarchical cluster overlaid on the T2 map. Voxels classified as “pathological” and “healthy” parenchyma are reported in red and yellow, respectively. **(F)** the results of gross anatomy. From the photograph of the brain section, the contour of the hemorrhagic lesion characterized by a linear shape that involves the left frontal lobe (white arrow) has been delineated (white dotted line), **(G)** histopathology results. The overall brain section obtained from the microscope with the hemorrhagic lesion shown in greater detail superimposed on the overall section is reported, and **(H)** a good agreement can be noted between the MRR classification and the lesion contour estimated from both gross anatomy and histopathology.

**Figure 4:**
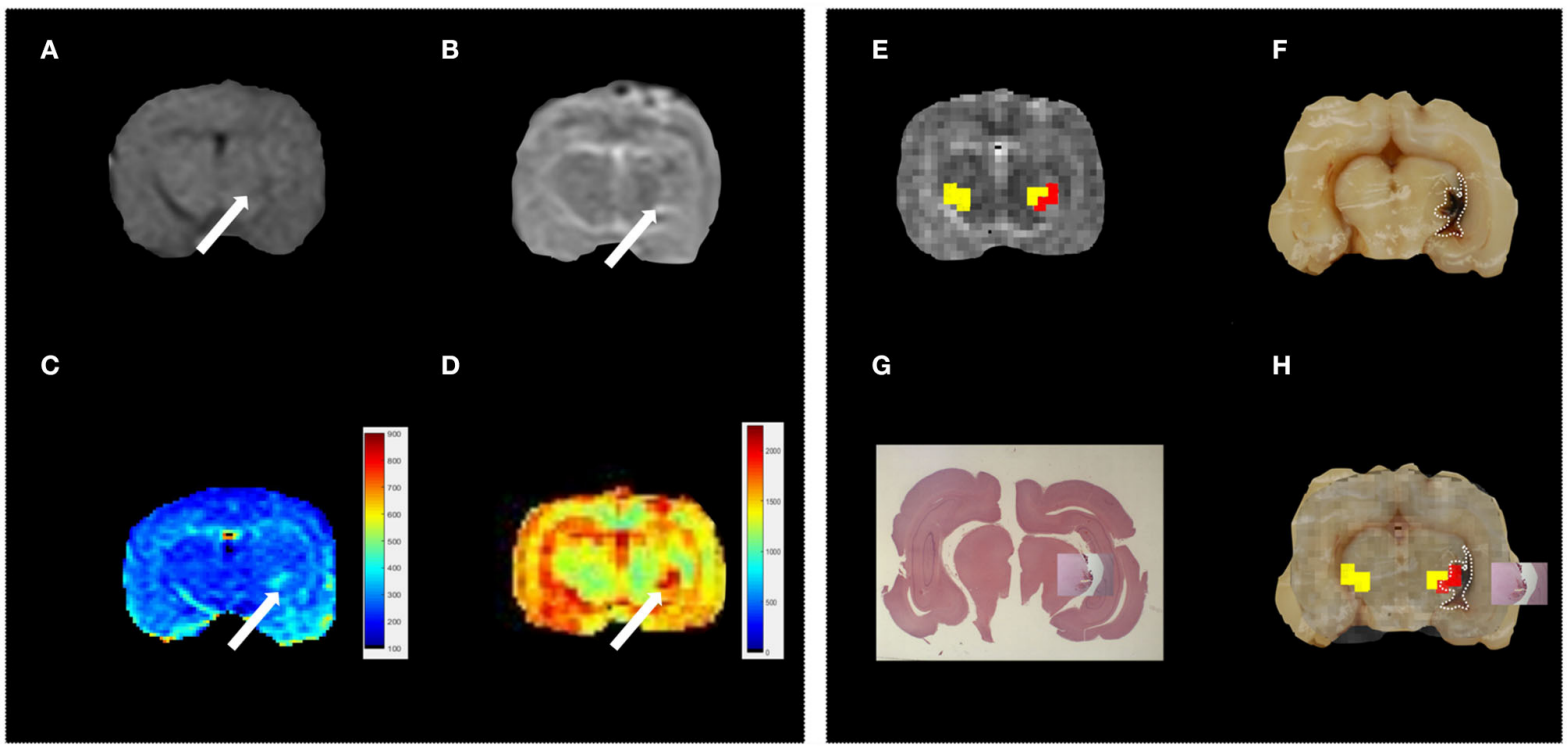
Standard vs. MRR analyses and histopathological validation. Example 3. In all panels, the left brain hemisphere is on the right part of the image. **(A)** T1w transverse image of the brain; the lesion appears ill-defined, hypointense, and irregular in the left thalamus (white arrow), **(B)** T2w transverse image of the brain. As in T1w, the lesion in the left thalamus appears ill-defined, irregular but hyperintense (white arrow), **(C,D)** T1 and T2 maps, respectively. A hyperintense and irregularly shaped area is visible in both maps (white arrow), **(E)** the output of the hierarchical cluster overlaid on the T2 map. Voxels classified as “pathological” and “healthy” parenchyma are reported in red and yellow, respectively, **(F)** the results of gross anatomy. From the photograph of the brain section, the contour of the hemorrhagic lesion that appears irregular in shape and involves the left thalamus (white arrow) has been delineated (white dotted line), **(G)** histopathology results. The overall brain section obtained from the microscope with the hemorrhagic lesion shown in greater detail superimposed on the overall section, and **(H)** the hemorrhagic lesion found by the MRR approach (red voxels) is in agreement with the gross anatomy. Voxels classified as pathological fall inside the true lesion contour. It can be noted that in **(F)** the hemorrhagic area appears more extended than the lesion histologically evident **(G)**. However, this can be due to postmortem blood spread in the space between the left hemisphere and the ipsilateral thalamus. The contour of the hyperintense area in both T1 and T2 maps is in very good agreement with the hemorrhagic area evident from the histopathological results where the blood spread disappeared after the formalin fixation.

First, the conventional MR protocol was tested to characterize the induced lesions. To this aim, we use both T1w and T2w images. A board-certified radiologist classified 23% of these lesions as “not visible,” 38% as “barely visible,” and 39% as “clearly visible.” As an example, in Figures 2–4A,B we report T1w and T2w images of three representative subjects. In 22% of the sample, the lesions were localized in the left frontal lobe (Figure 3), and in the remaining 78%, the lesions were localized in the Nucleus caudatus/left thalamus (Figures 2, 4). Then, the T1 and T2 maps were estimated. In this case, lesions were judged as “clearly visible” in most of the sample, namely 85% for T1 maps (see Figures 2–4C) and 93% for T2 maps (see Figures 2–4D). In the remaining 15 and 7%, for T1 and T2 maps, respectively, the lesion was not clearly detectable. In general, as can be seen in Figures 2–4C,D (white arrows), hyperintense lesions were observed with punctiform, linear, or irregular shapes. Lesion size varied across subjects.

### Tissue Classification

**Figure 5:**
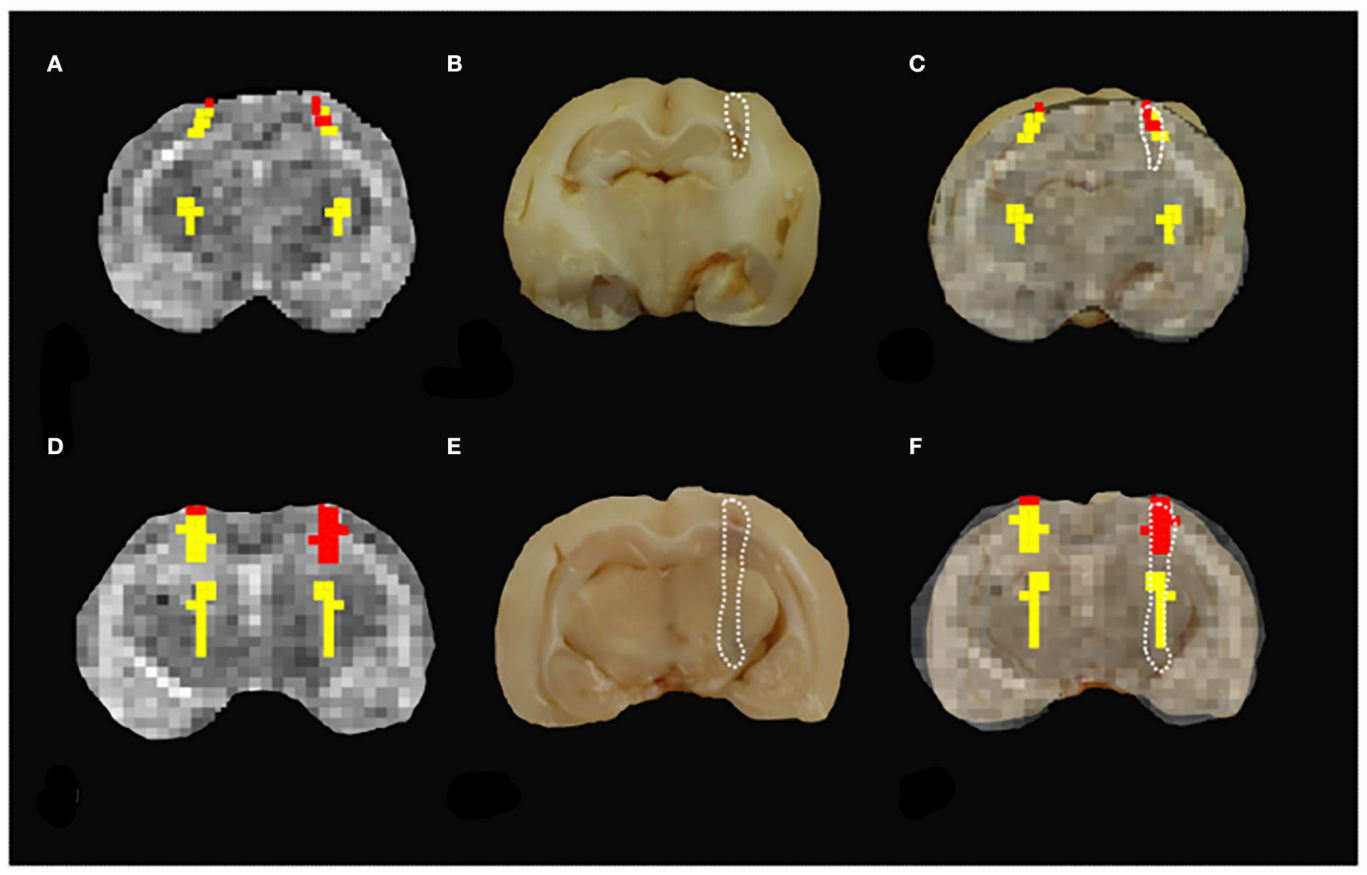
Misclassification results. **(A,D)** The results of MRR classification do not show a clear distinction between the two groups of pathological vs. healthy parenchyma since pathological tissue was found in the healthy hemisphere. **(B,E)** The hemorrhagic lesion contour (white dashed line) is estimated from the gross anatomy. **(C,F)** The comparison between the MRR classification and the true lesion contour shows that in these two cases the performance of the classifier is poor since both healthy (yellow) and pathological (red) voxels fall inside the lesion contour.

Eventually, hierarchical clustering was performed to classify brain tissues. As described in Materials and methods, a set of candidate voxels was selected based on T2 maps. We report in Supplementary Figure 2A (see Supplementary Material) the voxels selected (black dots) and their analogs (red dots) for the data in Figure 2. Based on their T1-relaxation signals (Supplementary Figure 2B), hierarchical clustering was performed. The final classification of healthy (H-yellow) and pathological (P-red) voxels is shown in Figures 2–4E overlaid on the T2 map (grayscale). Interestingly, in all reported cases, within the initially selected area, the hierarchical clustering identified a subset of “pathological” voxels. A possible reason is that the initial “suspected” area might contain both hemorrhagic and other types of “disturbed” tissue that seem to be distinguished in the classification output. Furthermore, since lesions were induced only in the left hemisphere, elements of the P class were not expected in the right hemisphere. This applied to the reported cases in Figures 2–4. However, sometimes the classification failed partially and labeled voxels in the healthy hemisphere as pathological, too (see for example Figure 5). This may be attributed to blood heparinization which may have led to a lack of a clear division between hemorrhage and brain parenchyma (see Discussion). These findings show that it is fundamental to validate accurately the proposed approach. To this aim, we assessed the classification performance by comparing these results with histopathology.

### Histopathology and Classifier Validation

**Figure 6:**
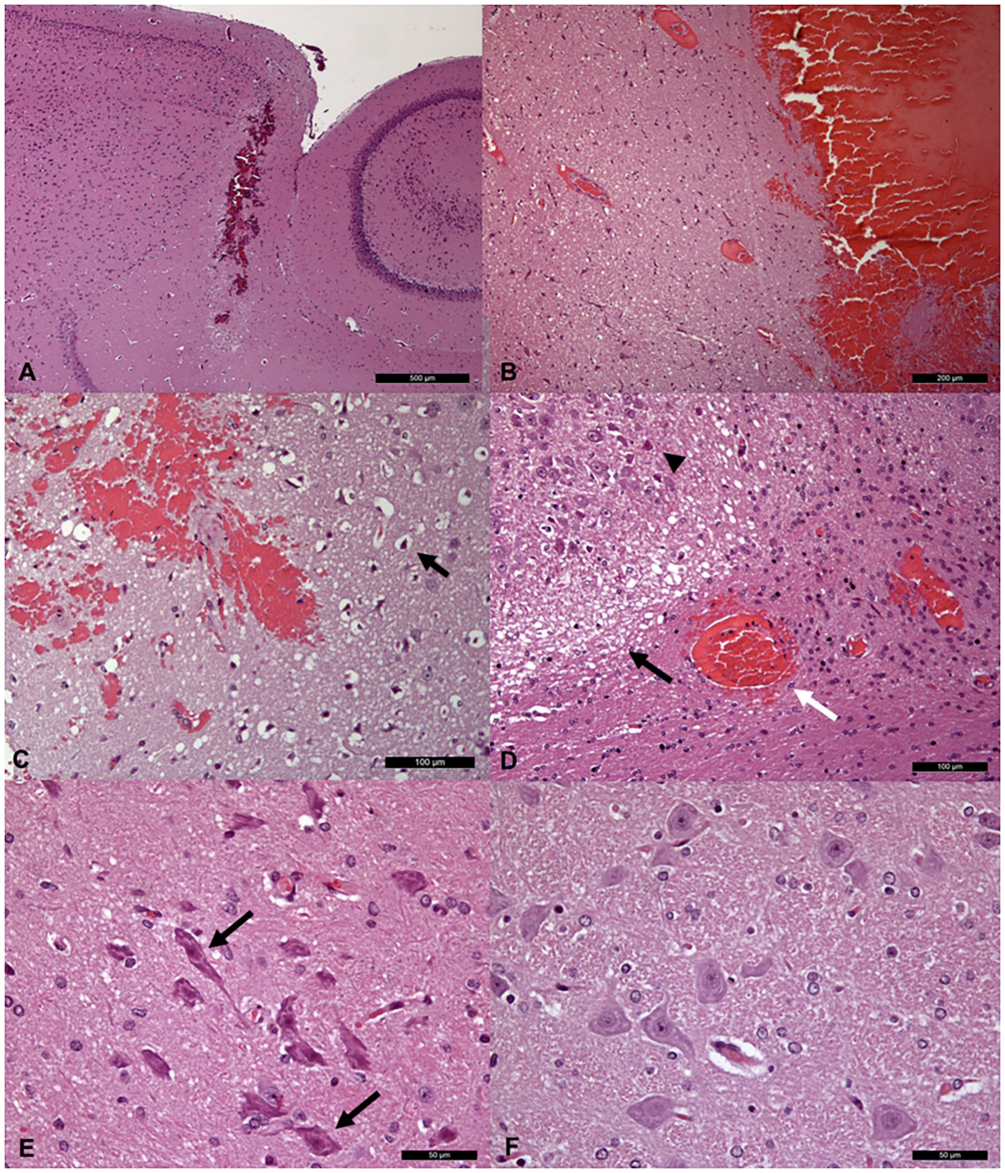
Histopathological sections **(A,B)** Low **(A)** and high **(B)** magnification of brain sections passing through the needle path, showing a linear hemorrhagic area. **(C,D)** Histological images of peri- hemorrhage neural tissue, showing variably intense, **(C)** gray (perinuclear halos; arrow), and **(D)** white matter edema (black arrow) and multifocal neuronal shrinkage (arrowhead). Red blood cells within the wall of a small vessel are also visible (white arrow). **(E)** Higher magnification of multifocal neuronal shrinkage (arrows) in peri-hemorrhagic tissue. **(F)** Histologically normal neurons in the contralateral area of the unaffected cerebral hemisphere.

**Figure 7:**
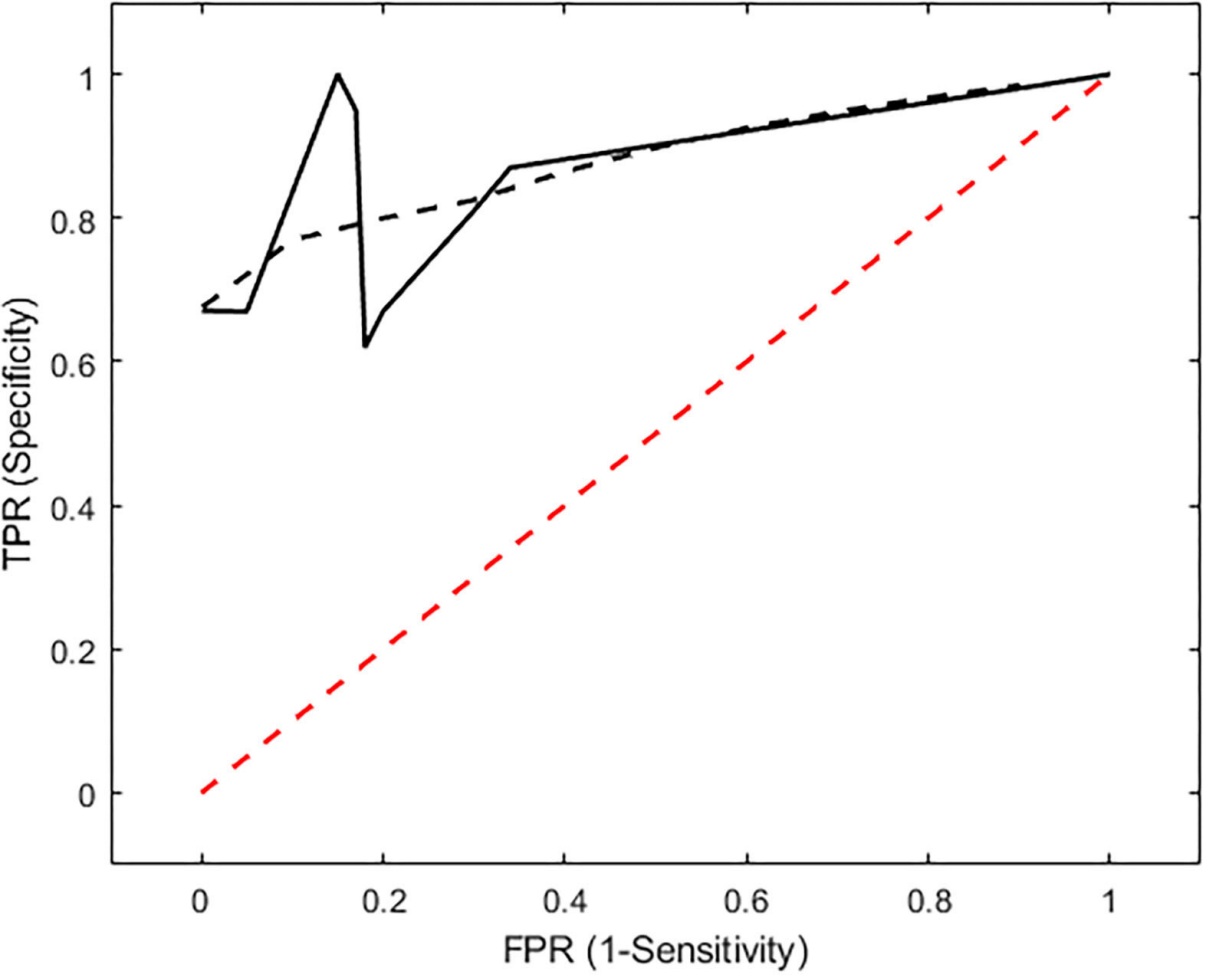
ROC analysis. The ROC curve shows that the classifier performance (black line) is beyond the chance level (red line). When the original data, reported in Table 2 are used, AUC = 0.88. To minimize the effects of TPR fluctuations due to the small sample size, we smoothed these values by means of a smoothing spline (black dashed line). In this case, AUC = 0.80. The AUC values, as compared to the thresholds reported in Materials and methods, place the performance of the classification at the “moderately accurate” level (AUC = 0.9).

The induced hemorrhages varied in size and shape, and a superficial, subdural, or subarachnoid collection of blood was also observed. The most frequently affected areas were the frontal cerebral cortex (gray and white matter), cingulate gyrus, and caudate nuclei. Serial sections allowed identification of the extent of the hemorrhagic area caused by the needle-induced direct trauma and the perilesional tissues not affected by the blood accumulation. The variation of intensity and extent of the lesion, due to the subjective tissue response, varied from one individual to another (Figure 6). The injected blood caused disruption of the surrounding neural tissue, termed the mass effect (Figures 6A,B). Fragmented nuclear debris was observed in damaged perihematomal tissue, in association with the presence of perihematomal edema, frequently characterized by gray matter vacuolization, and neuronal perinuclear halos (Figures 6C,D black arrow), and multifocal, mild dilation of perivascular (Virchow-Robin) spaces. Red blood cells within the wall of small vessels (intramural erythrocytes) and scattered swollen endothelial cells were also seen (Figure 6D white arrow). Also, multifocal cell shrinkage, with the presence of scattered hypoxic-ischemic neurons was detected in almost all cases (Figure 6D, arrowhead; Figure 6E, arrow). For comparison, neural tissues distant from the hematoma in the same section were examined, as well as the corresponding contralateral areas in the unaffected cerebral hemisphere, and no significant alterations were recorded. This excludes the occurrence of histologic artifacts due to brain fixation or manipulation (Figure 6F). The above aspects highlight the structural difference between the brain hemisphere with induced hemorrhage and the healthy one, confirming that the hyperintense areas on T1 and T2 maps were not artifactual in nature and correspond to real lesions. To validate the proposed approach, we compared the MRR with the histopathological results through a ROC analysis (see Materials and Methods). In Figures 2–4F, we show the lesion contour (dashed line) extracted from a photograph of the section of the brain immediately after the extraction, typically within 10–15 min after the end of the MRI session. This section was selected, as explained in Materials and methods, to match the MRI transverse plane where the relaxometry study was performed. Furthermore, the lesion contour was also obtained from the gross anatomy sections and microscopic analyses were performed 48 h after the brain was kept in formalin. The macroscopic image of the brain section with an inset from the microscopic analyses of the hemorrhages is reported in Figures 2–4G. As it can be noted in Figures 4H, 5, where the classification and anatomy results are superimposed, a good agreement between the gross anatomy (dashed) and MRR classified voxels (red) was obtained. However, as far as regards the cases shown in Figure 5, the classification seems to match only the initial part of the lesion (see Figure 5C) or half of it (Figure 5F). Now, to validate the MRR approach quantitatively (see Materials and methods), the TPR and FPR for each lesion were computed. The results reported in Table 2 show that the mean TPR was 0.76 and the mean FPR was 0.13, leading to an overall accuracy of 0.83. However, as it can be seen from Table 2, at FPR = 0 we obtained both high values of TPR (very close or equal to 1) as well as a couple of small values (around 0.3). TPR values around 1 are encouraging (no false positives and no false negatives) while the values around 0.3 show that in two cases the MRR finds some false negatives. To account for these fluctuations of the classifier, we performed the ROC analyses by using FPR = 0 the average value of TPR (0.67). We obtained AUC = 0.88 (Figure 7). Further, to estimate the effects of the ROC curve fluctuations on the AUC, likely due to the small sample size, we fitted the TPR values through a smoothing interpolating spline (Figure 7, dashed black line). We used the fitting curve toolbox in MATLAB with smoothing interpolating spline (smoothing parameter = 0.9995, see (39) for details). In this case, AUC = 0.80. Fluctuations seem to inflate the AUC. However, the obtained value still places the classifier in the moderately accurate class (0.7 < AUC <0.9, see Materials and methods). Of note, the autologous blood injection might provoke a tissue injury surrounding the blood hemorrhage that can hardly be distinguished from the blood. This might explain why the selected initial areas were often smaller than the final classified lesion, and why the classification failed partially in some cases (see Figure 5). However, it is important to stress that the histopathological analysis can clearly distinguish between these cases. Thus, the final accuracy and sensitivity of our approach account for these confounding effects.

**Table 2:** ROC analyses. TPR, FPR, and accuracy are reported for each rabbit.

**Rabbit**	**True Positive Rate**	**False Positive Rate**	**Accuracy**
1	0.87	0.34	0.77
2	0.62	0.18	0.74
3	1	0	1
4	0.81	0.3	0.76
5	0.67	0.05	0.89
6	0.67	0.2	0.74
7	1	0.15	0.92
8	0.34	0	0.67
9	1	0	1
10	0.95	0.17	0.88
11	1	0	1
12	0.3	0	0.65
13	0.91	0	0.96
Mean	0.76	0.12	0.83

## Discussion

In this study, we proposed a combined T1–T2 MRR approach to characterize mild brain hemorrhages in a rabbit model. The developed technique supports a diagnosis at the subject level; it was compared to conventional MRI and validated through histopathology. The proposed approach, being optimized for a low field scanner routinely available in veterinary facilities, has the advantage of a potential direct translation into clinical practice. Our findings suggest that T1/T2 relaxometry, although only indirectly linked to the blood content, provides a higher contrast as compared to conventional imaging in identifying brain lesions (25). We tested our approach on induced lesions obtained by injecting a minimal amount of blood to create a reproducible and lateralized damage. This allowed us to compare the injured and healthy parenchyma within a single MR scan. We do not expect any sex-related bias (e.g., due to the size or anatomy). This approach is in line with previous works. For example, in a previous study (40), the authors injected autologous blood in the right hemisphere of rat brains to evaluate the effects of experimental intracerebral hemorrhage on brain tissue injury and recovery. They reported that MR relaxometry can be promising to observe the lesion progression. From a purely technical point of view, it was easier to perform this kind of analysis with T1 and T2 sequences. There was no need for external technical support to select the proper parameters to acquire the relaxometry data. Further, it was possible to manually select the TR or TE values to acquire MRR images. The same did not apply to T2^*^ sequences, with our system. This aspect was in line with our aim to provide a system readily available for most practitioners. The proposed approach combined both T1 and T2 information: the highest contrast in the T2 map was exploited to select the initial ROIs, while the hierarchical clustering was driven by the T1-based signal recovery. In fact, in terms of qualitative evaluation, our data show that the lesions were always more evident in T2 than in T1 maps. Moreover, the adoption of the T1 signal to drive the classification is in agreement with the literature, where T1 relaxometry has proven useful to assess brain parenchyma (28, 40), even with low filed scanners (22, 41). Nevertheless, this choice leads to a longer acquisition time, which is one of the main drawbacks of the proposed approach. Nowadays, advanced MR techniques, such as SWI, MT, and T2^*^ based relaxometry, are being developed to identify brain hemorrhages. It is important to put the MRR novelties and limitations within the context of these advanced techniques. The SWI, with its use of both phase and magnitude data, is highly sensitive to paramagnetic susceptibility effects due to paramagnetic substances, such as deoxygenated blood, blood products, iron, and calcium (42–44). This allows SWI to identify brain hemorrhage, mineralization, venous abnormalities, and tumor neovascularity, thus making it a potential clinical technique for many intracranial pathologies. However, the susceptibility effect requires non-refocused GE techniques using long TE, short flip angles, and more importantly high field strengths. Further, T2^*^ can detect brain hemorrhages, too, by exploiting the local magnetic field inhomogeneities induced by paramagnetic deoxyhemoglobin, methemoglobin, or hemosiderin present in the brain parenchyma (45–47). These sequences, in some cases, seem to outperform the FLAIR sequence (48). However, there are several limitations of these sequences, especially at low field (in our case B0 = 0.25 T). The signal-to-noise ratio is low, and any para/ferromagnetic material or air-brain interface might result in an artifact related to susceptibility (this effect is even stronger in high fields). This can impair the interpretation or even render the sequence non-diagnostic, as occurred in (49, 50). In addition, the susceptibility artifact can “bloom,” making the size or internal characteristics of a lesion difficult to determine. In this case, it is difficult to estimate the age of a hemorrhagic lesion (51). Subtle parenchymal changes should be interpreted with caution and always be compared to sequences with higher soft-tissue contrast and signal-to-noise ratio (48). In regards to FLAIR sequences, it might have been interesting to compare them with our MRR results. At this stage, we did not adopt these sequences since the fluid attenuation might have affected MR signals within and around the provoked lesion. However, we acknowledge that we did not investigate these aspects in detail and this is certainly an interesting topic for future development. Magnetization transfer (MT) techniques refer to the exchange of magnetization between hydrogen nuclei bound to water and those bound to semi-solid macromolecules. The latter population, while significant in number, lose transverse magnetization due to T2 decay on a time scale of ~10 μs. This time is too short for direct observation using a clinical MRI scanner. When a high field scanner is available, nuclei bound to large molecules will result in a useful tissue contrast. This allows for investigating of brain development, aging, and disease (52–54). However, this approach has seldom been used for routine clinical neuroimaging. The scans take longer to acquire than standard T1/T2w imaging, and the most easily acquired image contrast, based on magnetization transfer ratios, appears qualitatively similar to T1w scans. To provide quantitative imaging, advanced MT techniques require the adoption of complex biophysical models. These rely on MR properties (relaxation and exchange rates, pool sizes, etc.), rather than biochemical, cellular, and structural characteristics of brain tissue (52). In this scenario, it must be acknowledged that the above techniques are potentially more sensitive than the MRR approach in characterizing brain hemorrhages in terms of tissue structure. However, a common feature of these approaches is the need for a high field scanner for advanced sequences, appropriate SNR, and complex hardware for the acquisition. For this reason, even when a high field scanner is available, these techniques are seldom used in clinical practice (52). This is even more stringent in the clinical context of veterinary medicine, where high field scanners are not typically available, and the diagnosis is still based on conventional MRI. For this reason, the novelty of this work consists in developing an advanced MR technique that can be used in the clinical field with a low field scanner. However, we acknowledge that the lack of a comparison with T2^*^, FLAIR, and SWI is a limitation of this study. In terms of classification, we adopted hierarchical clustering which has several advantages as compared to other techniques, e.g., K-means. The number of classes does not need to be specified in advance and the progression of the clustering can be evaluated at different levels. This approach has been previously applied in the histopathological field to distinguish between different tissue features (55–57) and lesions characterized by different patterns (58–60). In the case of mild lesions, such identification with standard MRI techniques can be very challenging, and any advanced analysis technique must be validated. To this aim, the classification performance was directly addressed through the comparison with histopathological analyses. The histopathological findings show that in acute human spontaneous intracerebral hemorrhages, the primary tissue damage is followed by secondary lesions in which cellular and neuroinflammatory changes are poorly defined. This might be due to mass effect and mechanical disruption. These lesions are in line with those induced by injecting blood in different animal models (61). Most of the studies published on this topic refer to 24/48 h postmortem histological changes (62, 63), especially focusing on the time and mass effect of the lesion, cerebral edema, and ischemic cell changes affecting the adjacent neural tissue. Consistently, in all our cases, the histopathological examination on fixed tissue, 1 h after the MRI session, showed initial alterations such as peri-hemorrhage slight edema as well as mild vascular and neuronal degeneration. These findings were crucial to confirming our MR tissue classification. In fact, the lesion contours extracted using the gross anatomy and histopathological images showed a good performance in the MRR classification. However, in some cases, the adopted approach failed partially to identify the lesion. This could be due to a small amount of blood that (likely because of heparinization), infiltrated slightly the neural tissues around the line of needle insertion during the injection. Such a small blood infiltration in the surrounding healthy parenchyma could make the MRR clustering unable to distinguish the boundary between real hemorrhage-healthy tissues. This distinction appears evident from the histopathology after the brain fixation. This hypothesis related to heparinization is supported by findings of a prior study, where, unlike what has been herein described, the authors used autologous non-heparinized blood injection into cerebral parenchyma for their experiment and observed a clear distinction between hemorrhagic tissue and parenchyma (61). In general, caution must be used in comparing the histopathological and MRI analyses for several reasons, including different slice thickness on MRI images and gross anatomy, potential misalignment, and the effect of formalin fixation. Specifically, it has been reported that there is a reduction in tissue volume of ~33% after formalin fixation and paraffin embedding (64). This may have influenced the data comparison, since after tissue fixation the size of the original lesion investigated with relaxometry may have changed, thus relying on a possible bias during results data co-registration and agreement quantification. Nevertheless, the obtained sensitivity and specificity show that MRR can be considered moderately accurate. The current results pave the way for future artificial intelligence (AI) based techniques which are being developed to assist physicians in improving their diagnosis (65). In this scenario, after being trained, a classification algorithm automatically maps the observations to a set of classes. Our work can be considered seminal for future AI applications validating a combined T1/T2 MRR hierarchical clustering for the classification step (66, 67). At the current stage, in our approach, the training is missing, and the proposed procedure is semi-automatic relying on a manual selection of candidate ROIs. However, the algorithm training requires datasets of a larger size than our current sample. Therefore, it represents a future development of this technique where part of the data will train the classifier, and the remaining will be automatically classified (68, 69).

## Conclusion

To conclude, this work shows that an advanced MRR protocol is promising even with a low field scanner (0.25 T) in a clinical setting. The proposed approach, validated with histopathology, seems to outperform conventional MRI in accurately classifying blood products in brain parenchyma. In future studies, these findings must be confirmed on larger samples, and new acquisition sequences must be optimized to reduce the acquisition time. In this way, the MRR approach might become an automatic classification tool available on routine scanners for veterinary medicine.

## Data Availability Statement

The original contributions presented in the study are included in the article/Supplementary Material, further inquiries can be directed to the corresponding authors.

## Ethics Statement

The animal study was reviewed and approved by National Committee for Animal Welfare, with the protocol N° 726/2019-PR.

## Author Contributions

FD, MV, LDS, and FP: conceptualization, writing—original draft, resources. FD, MV, LDS, RT, IC, AP, and FP: data curation. FD, MV, LDS, RT, IC, AP, MR, and FP: formal analysis. MV and FP: funding acquisition and project administration. FD, MV, LDS, RT, AP, MR, and FP: investigation. FD, MV, LDS, RT, IC, MR, IF, and FP: methodology. FD and FP: software. MV, LDS, and FP: supervision. FD, MV, LDS, RT, IC, AP, ER, NF, MR, IF, and FP: validation. FD, MV, LDS, RT, IC, AP, ER, NF, MR, and IF: visualization. FD, MV, LDS, RT, IC, AP, MC, ER, NF, MR, and IF: writing—review and editing. All authors contributed to the article and approved the submitted version.

## Funding

This study was partially funded by the Demetra Project (Departments of Excellence 2018-2022, CUP _C46C18000530001), funded by the Ministry of Education, University and Research. The funders had no role in study design, data collection, analysis, decision to publish, and preparation of the manuscript. This study was partially funded by the PON research project 2014-2020 (CCI 2014IT6M2OP005).

## Conflict of Interest

The authors declare that the research was conducted in the absence of any commercial or financial relationships that could be construed as a potential conflict of interest.

## Publisher's Note

All claims expressed in this article are solely those of the authors and do not necessarily represent those of their affiliated organizations, or those of the publisher, the editors and the reviewers. Any product that may be evaluated in this article, or claim that may be made by its manufacturer, is not guaranteed or endorsed by the publisher.

## Supplementary Material

Supplementary Figure 1 Technical procedure to induce hemorrhages. **(A)** The brain calvarial intersection between the coronal and the caudal suture is used as an anatomic landmark (black arrow), **(B)** the 2-mm diameter hole performed with a surgical drill 3–5 mm caudally and on the left of the coronal suture, and **(C)** the final location to inject the autologous blood and induce the cerebral hemorrhage.

Supplementary Figure 2 The analysis pipeline. Here we schematized the steps involved in the analysis from the initial selection of areas to the final classification output and ROC analyses.

Supplementary Figure 3 The procedure adopted for the image co-registration. **(A)** The brain cut was performed to obtain a plane passing through the needle injection, clearly visible, with the same inclination estimated from the MR session, and perpendicular to the brain midline. **(B)** The resulting cut. **(C,D)** From the macroscopic image (MI) several histological samples were extracted to characterize the structure of the internal lesion, (see the inset) to draw the final lesion contour. **(E)** The obtained lesion contour is overlaid (white line) to the MI. **(F)** The T2w image of the same subject that will be used in the co-registration. **(G)** The adopted parameters are manually optimized by the radiologist and pathologist. In the upper part of the panel, we report the parameters used for the similarity matrix estimation. In the lower part, the ones related to the non-rigid transformation. **(H)** Top, the resulting similarity matrix. Bottom, the obtained deformation fields for the non-rigid transformation. **(I)** The subsequent steps of co-registration. The MI is reported in magenta while the T2w is reported in gray. A good alignment between the two images after the non-rigid transformation is noted. Once the images are aligned, the true lesion contour (white line) was overlaid on the T2w images.
