# Supplementary figures and images for: A Magnetic Resonance-Relaxometry-Based Technique to Identify Blood Products in Brain Parenchyma: An Experimental Study on a Rabbit Model
Source: Front Vet Sci. 2022 May 31;9:802272. doi: 10.3389/fvets.2022.802272 (PMC9195168; doi:10.3389/fvets.2022.802272)

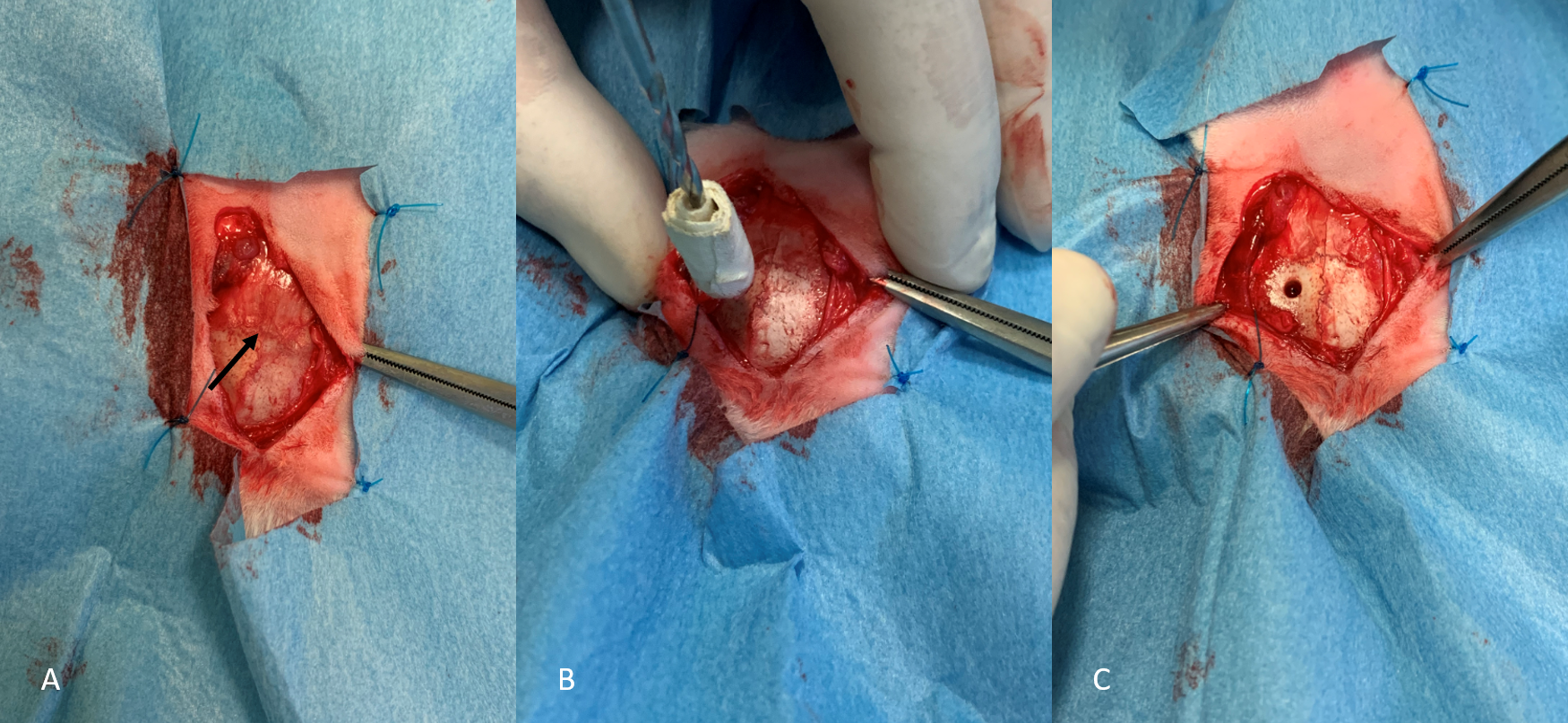

Supplement: Supplementary Figure 1 — Technical procedure to induce hemorrhages. (A) The brain calvarial intersection between the coronal and the caudal suture is used as an anatomic landmark (black arrow), (B) the 2-mm diameter hole performed with a surgical drill 3–5 mm caudally and on the left of the coronal suture, and (C) the final location to inject the autologous blood and induce the cerebral hemorrhage. [file Image_1.TIF]

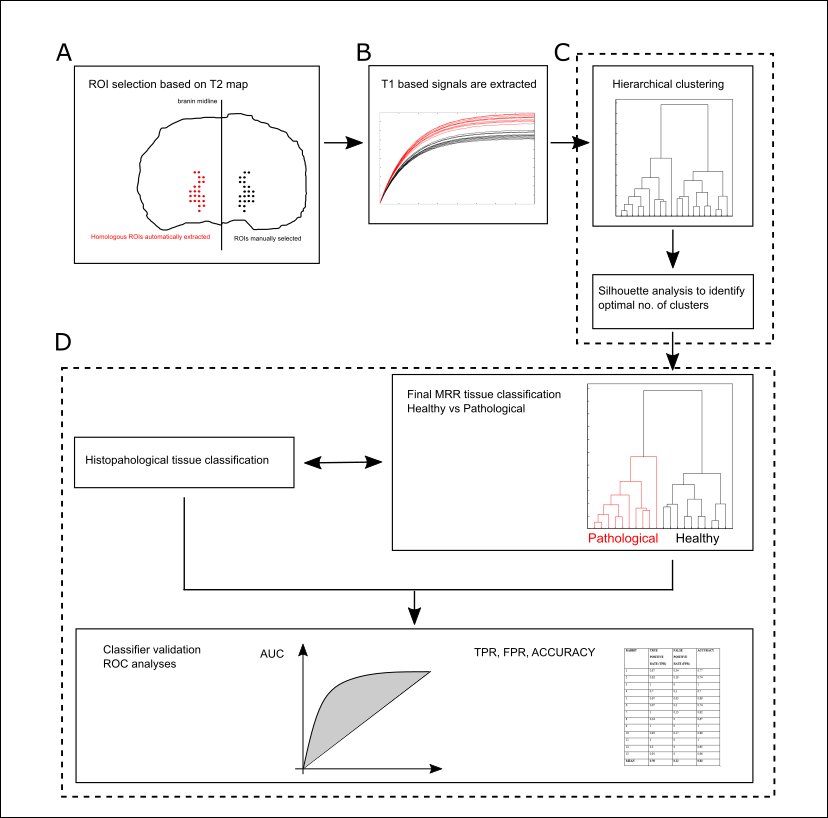

Supplement: Supplementary Figure 2 — The analysis pipeline. Here we schematized the steps involved in the analysis from the initial selection of areas to the final classification output and ROC analyses. [file Image_2.TIF]

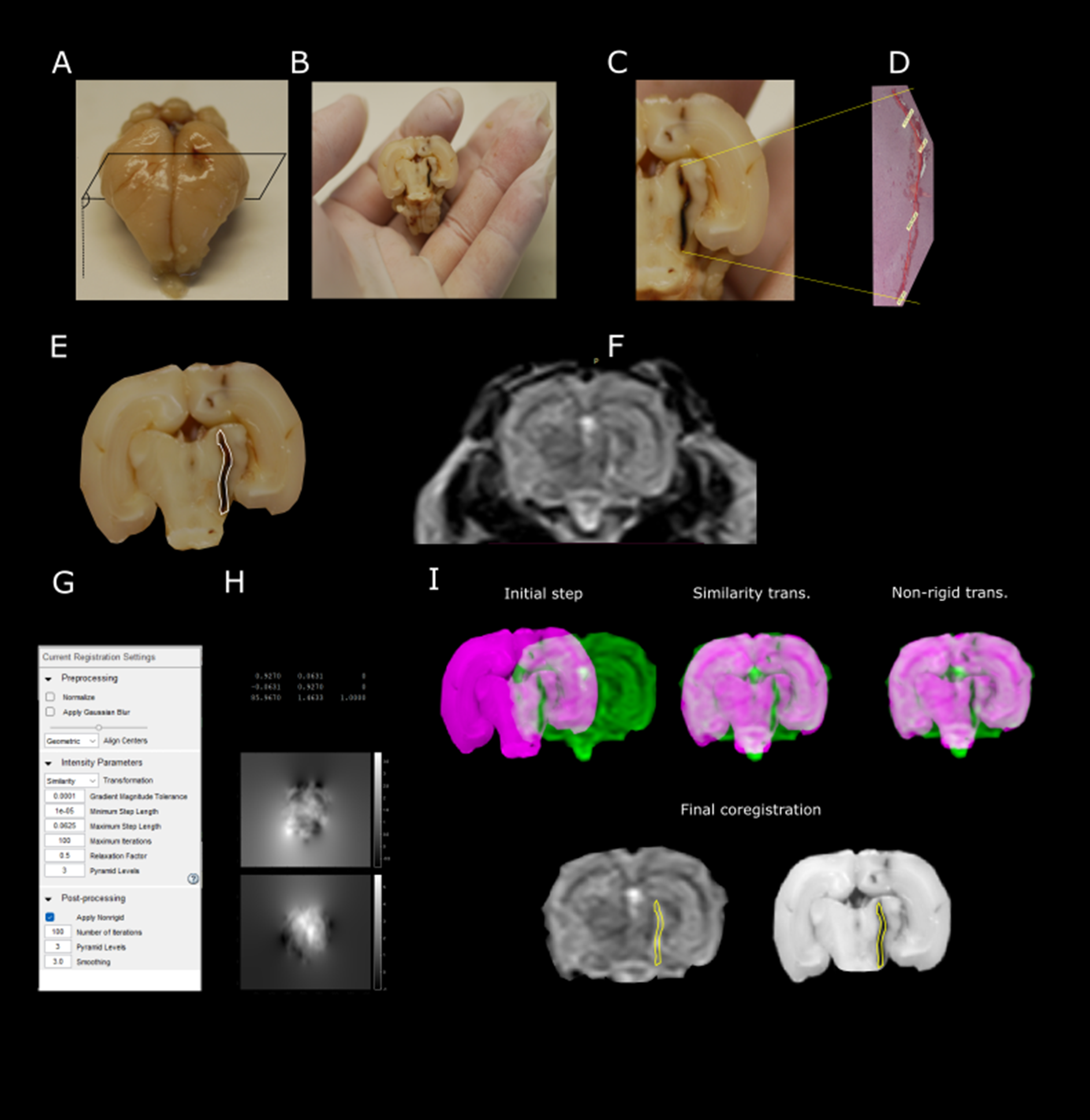

Supplement: Supplementary Figure 3 — The procedure adopted for the image co-registration. (A) The brain cut was performed to obtain a plane passing through the needle injection, clearly visible, with the same inclination estimated from the MR session, and perpendicular to the brain midline. (B) The resulting cut. (C,D) From the macroscopic image (MI) several histological samples were extracted to characterize the structure of the internal lesion, (see the inset) to draw the final lesion contour. (E) The obtained lesion contour is overlaid (white line) to the MI. (F) The T2w image of the same subject that will be used in the co-registration. (G) The adopted parameters are manually optimized by the radiologist and pathologist. In the upper part of the panel, we report the parameters used for the similarity matrix estimation. In the lower part, the ones related to the non-rigid transformation. (H) Top, the resulting similarity matrix. Bottom, the obtained deformation fields for the non-rigid transformation. (I) The subsequent steps of co-registration. The MI is reported in magenta while the T2w is reported in gray. A good alignment between the two images after the non-rigid transformation is noted. Once the images are aligned, the true lesion contour (white line) was overlaid on the T2w images. [file Image_3.TIF]
